# Identification of a crenarchaeal orthologue of Elf1: implications for chromatin and transcription in Archaea

**DOI:** 10.1186/1745-6150-4-24

**Published:** 2009-07-29

**Authors:** Jan-Peter Daniels, Steven Kelly, Bill Wickstead, Keith Gull

**Affiliations:** 1 Sir William Dunn School of Pathology, University of Oxford, South Parks Road, Oxford, OX1 3RE, UK; 2 Centre for Mathematical Biology, University of Oxford, 24-29 St Giles', Oxford, OX1 3LB , UK; 3 Oxford Centre for Interactive Systems Biology, Department of Biochemistry, University of Oxford, South Parks Road, Oxford, OX1 3QU, UK

## Abstract

The transcription machineries of Archaea and eukaryotes are similar in many aspects, but little is understood about archaeal chromatin and its role in transcription. Here, we describe the identification in hyperthermophilic Crenarchaeota and a Korarchaeon of an orthologue of the eukaryotic transcription elongation factor Elf1, which has been shown to function in chromatin structure maintenance of actively transcribed templates. Our discovery has implications for the relationship of chromatin and transcription in Archaea and the evolution of these processes in eukaryotes.

This article was reviewed by Chris P. Ponting and Eugene V. Koonin.

## Findings

Despite their prokaryotic morphology, Archaea are more similar to eukaryotes in their mechanisms of copying and expression of their genetic information than to bacteria [1]. With the recent description of an RPB8 orthologue (RpoG) in hyperthermophilic Crenarchaeota and a Korarchaeon [2] and demonstration of its constitutive incorporation into archaeal RNAP [3], homologues of all of the twelve DNA-dependent RNA polymerase (RNAP) eukaryotic core subunits have been identified in Archaea [4]. The structure of the archaeal RNAP closely resembles the eukaryotic RNAPII [5]. Eukaryotic transcription furthermore depends on accessory factors aiding initiation. Of those, homologues of eukaryotic basal transcription initiation factors TBP (TATA-binding protein), TFIIB (transcription factor II B) and of the α-subunit of TFIIE are found in Archaea (TBP, TFB and TFE) [1].

In eukaryotes, transcription elongation factors assist RNAP in overcoming pausing and arrest on the template [6]. TFIIS releases RNAPII from transcriptional arrest by supporting RNA transcript cleavage, whereas the yeast DSIF complex consisting of Spt4 and Spt5 (bacterial homologue NusG) is thought to belong to the class of chromatin elongation factors that affect RNAP transcription through chromatin [7]. The archaeal TFIIS-homologue TFS appears to operate in an equivalent manner to eukaryotic TFIIS [1]. Also, an orthologue of Spt5/NusG and a protein with sequence and structural similarity to Spt4 have been identified in Archaea, further supporting the ancestral link between archaeal and eukaryotic transcription [1].

Elf1 is a transcription elongation factor that has recently been identified and characterized in *Saccharomyces cerevisiae *in a screen for mutations that cause synthetic lethality with mutations in other genes coding for transcription elongation factors [8]. A role for Elf1 in transcription elongation was demonstrated by genetic interaction with several transcription elongation factor genes, including those coding for TFIIS, Spt4 and Spt5. Elf1 is recruited to regions of active transcription [8]. Transcription initiation from a gene-internal site in *elf1Δ *cells and the production of short transcripts in an *elf1Δ hir1Δ *background strongly suggested that Elf1 acts by maintaining the chromatin structure of active transcription units [8].

During a sensitive sequence-similarity search for transcription elongation factors in an evolutionary wide range of organisms, we noticed high-scoring hits for Elf1 in a subset of archaeal predicted proteomes. Consequently, a thorough search of more archaeal genomes was initiated. A multiple sequence alignment of *Homo sapiens *Elf1 (NP_115753.1) and *S*.*cerevisiae *Elf1 (NP_012762.1) was generated using MAFFT [9] and trimmed to well aligning sequence blocks. The alignment was then utilized to build a profile-hidden Markov model [10], which was queried against the predicted proteomes of 48 archaeal and 28 eukaryotic organisms with a wide evolutionary diversity (Additional file 1, Tables S1 and S2). Hits with expectation values lower than a threshold of 10^-3 ^were selected and aligned. The alignment was trimmed to aligned sequence containing no more than 50% gaps and sequences were removed so that no sequence pairs with an identity higher than 95% remained in order to prevent sequence bias. A new hidden-Markov model was built and the whole process was repeated iteratively until no new sequences could be identified [11]. This method provides a sensitive and high-quality dataset of Elf1 homologues.

**Figure 1:**
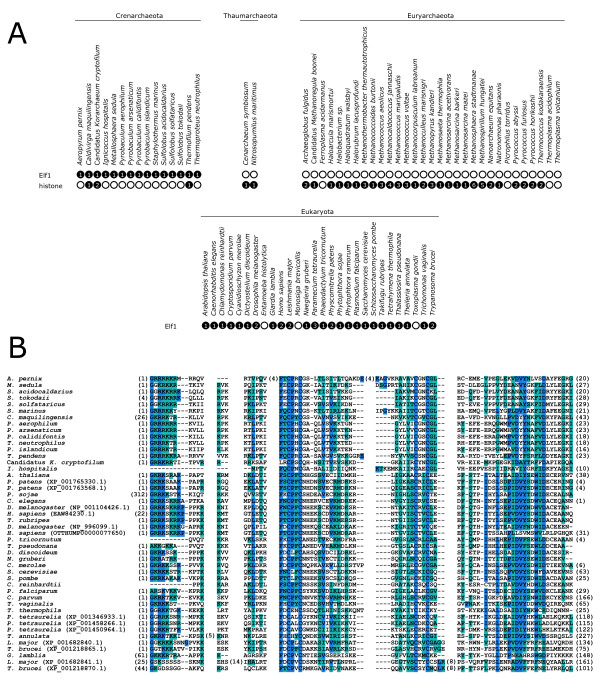
**A. Organisms with Elf1 and histone orthologues identified in this study**. The number in the black circle indicates the number of Elf1 and histone orthologues found. An empty circle indicates that no orthologue was detected. Histone searches were not done in for eukaryotic organisms. **B**. Alignment of Elf1 orthologues identified in this study. Organisms are indicated on the left. Numbers of trimmed residues are indicated in brackets at their respective position. Residues that are identical or similar to the consensus are shown with a blue or cyan background, respectively.

In this way, we found 46 sequences from 14 archaeal and 25 eukaryotic organisms (Figure 1A; Additional file 1, Tables S3 and S4). All the Elf1 homologues identified contain a C4-zinc finger signature and most sequences are characterized by a stretch of up to seven consecutive basic amino acids at their N-terminus (Figure 1B). All Archaea and most eukaryotes with an Elf1 only contain a single gene, although some eukaryotic organisms have lineage-specific gene duplications (Figure 1A). Importantly, Elf1 homologues were restricted to a distinct subset of the archaeal predicted proteomes. Elf1 was identified only in hyperthermophilic Crenarchaeota and the Korarchaeon Candidatus *Korarchaeum cryptofilum*. It was found in none of the predicted proteomes from Euryarchaeota or in the mesophilic marine group I *Cenarchaeum symbiosum *and *Nitrosopumilus maritimus *[12-14] (now classified as belonging to the Thaumarchaeota) [15]. This demonstrates that these organisms either do not have Elf1 orthologues or that homologues in these lineages are very divergent from both crenarchaeal and eukaryotic versions.

The only hyperthermophilic Creanarchaeon without a hit for Elf1 in the profile-hidden Markov model-based searches of its predicted proteome was *Thermofilum pendens*. In order to ensure that Elf1 orthologues were not missed in our searches because they had not been annotated in the predicted proteomes, we conducted tBLASTn [16] queries of the archaeal genome sequences with all 14 identified archaeal Elf1 proteins and gene order analyses using the UCSC Archaea genome browser [; Additional file 1, Table S5; [17]]. By this approach, Elf1 was found in all hyperthermophilic Crenarchaeota, including *T*. *pendens*, and Candidatus *K*. *cryptofilum*, but not in any Euryarchaeota, *C*.*symbiosum *or *N*.*maritimus *(Figure 1). Failure to detect an Elf1 orthologue in *C*.*symbiosum *and *N*.*maritimus *is in agreement with their phylogenetic position in a separate archaeal phylum, the Thaumarchaeota, as recently proposed [15]. Although Pfam family PF05129.5 describes many of the orthologues relationships identified in this work, we chose our iterative hidden Markov model approach, because this enabled us to define a species set and gathering threshold which would be both sensitive and selective for this specific task. Comparison confirms that our method was more selective (Additional file 1, Fig. S1). Other iterative procedures, such as PSI-BLAST [16], may achieve similar results, however.

Interestingly, this phylogenetic distribution is identical to that of RpoG, the divergent archaeal orthologue of the eukaryotic RNAP subunit RPB8 [2]. The same group of archaeal organisms that contain the full set of all twelve RNAP subunits also has an orthologue of the eukaryotic transcription elongation factor Elf1. This raises interesting questions as to whether and how Elf1 functions in archaeal transcription and whether it interacts with particular subunits of RNAP. Considering the function of yeast Elf1 in the maintenance of chromatin structure on actively transcribed genes [8], the issue arises as to how chromatin is constructed in Archaea. In eukaryotic chromatin, nucleosomes consist of the highly conserved histone proteins H2A, H2B, H3 and H4. Archaeal histones have been found in many euryarchaeal organisms and *Cenarchaeum *[18] and have recently also been described in *T*. *pendens *and *Korarcheum *[19,20]. In order to revisit this question in the light of new sequence data, we built a profile-hidden Markov model for archaeal histones. In addition to *T*. *pendens *(ABL77757.1), and Candidatus *K*.*cryptofilum *(ACB06807.1 and ACB07883.1), we were able to also identify a histone homologue in *Caldivirga maquilingensis *(ABW02527.1) (Figure 1A; Additional file 1, Figure S2). Thus, only some of the archaeal genomes in which we identified an Elf1 orthologue also code for histones. It is therefore likely that the function of archaeal Elf1 is independent of histone-containing chromatin.

Other, non-histone chromatin proteins have also been identified in Archaea. Alba is found in Crenarchaeota and a subset of Euryarchaeota [18], while Sul7d is restricted to *Sulfolobus *[21]. In eukaryotes, the transcription of a template is affected by its chromatin state, which can be regulated by post-translational modifications of terminal tails that protrude from the histone core. In Archaea, chromatin can also impair transcription, as nucleosomes slow RNAP down in vitro [22]. Archaeal histones lack protruding tail sequences [18], but Alba can be post-translationally acetylated at a lysine residue. Deacetylation of Alba by the conserved deacetylase Sir2 increases the DNA-binding affinity of Alba and impairs transcription in vitro [23]. Thus, it appears that Archaea have the ability to regulate transcription at the chromatin level in a similar way to eukaryotes. Our identification of an Elf1 orthologue in Archaea indicates that these mechanisms might be even more similar than previously thought, despite major differences in the composition of the chromatin template. It thus seems possible that a common ancestor of eukaryotes and Archaea already employed chromatin structure-modulating factors to regulate transcription. It will be very exciting in the future to learn about a role of archaeal Elf1 in transcription and how its function can be applied to non-histone chromatin templates.

## Competing interests

The authors declare that they have no competing interests.

## Authors' contributions

JPD and SK conceived the study and carried out the analysis for Elf1 and histones, respectively. All authors designed the experiments and analysed the data. JPD drafted the manuscript which was read and approved by all authors.

## Reviewers' comments

### Reviewer 1

Dr. Chris Ponting, University of Oxford, UK

#### Reviewer's comments

This manuscript describes a thorough analysis and a compelling argument for the existence of archaeal Elf1 orthologues. Whilst these findings are relatively straightforward to derive using standard database search tools, including PSI-BLAST and the authors' method of choice HMMer, the manuscript provides a detailed case for the evolutionary and functional importance of these findings. One addition that I would recommend is for the authors to acknowledge that Pfam has already collated many or all of these orthology relationships (see ) and differences from this Pfam family need to be stated explicitly.

#### Author's response

We probably didn't make the relationship between the Pfam Elf1 model and our HMM sufficiently clear in the manuscript. As mentioned, there already exists an HMM for Elf1-like proteins provided by Pfam. However, because Pfam families are built with a slightly different aim from that of the paper, we chose to iteratively build a bespoke HMM, because this enabled us to define a species set and gathering threshold which would be both sensitive and selective for this specific task.

Attached (Additional file 1, Figure S1) is a comparison of the results of searches using our HMM with those performed with the Elf1 HMM provided by Pfam (PF05129.5). These data demonstrate that, while both models identify the true Elf1 homologues in the organisms searched (labelled in red), our HMM provides a greater ability to distinguish between positives (red) and negatives (black).

#### Reviewer's response

I support publication of this manuscript with this additional Supplemental Figure.

### Reviewer 2

Dr. Eugene V. Koonin, NCBI/NLM/NIH, USA

#### Reviewer's comments

This is a straightforward but quite valuable paper that extends the homologous relationship between the archaeal and eukaryotic transcription machineries to include the crenarcaheal orthologs of the eukaryotic chromatin remodeling factor Elf1. The results are valid and complete (see comments below for some possible extensions), and also rather unexpected because Elf1 is thought to function in the maintenance of eukaryote-specific chromatin structure. This paper shows that there might be even more functional similarity between the process of transcription in eukaryotes and some archaea (in particular, Crenarchaeota) than currently thought.

## Supplementary Material

Additional file 1 **Supplementary tables and figures.** Click here for file
