# Supplementary material for: Identification of a crenarchaeal orthologue of Elf1: implications for chromatin and transcription in Archaea
Source: Biol Direct. 2009 Jul 29;4:24. doi: 10.1186/1745-6150-4-24 (PMC2732611; doi:10.1186/1745-6150-4-24)
Supplement: Additional file 1 — Supplementary tables and figures. [file 1745-6150-4-24-S1.pdf]

SUPPLEMENTAL MATERIAL

FOR

Discovery note

Identification of a crenarchaeal orthologue of Elf1: implications for chromatin and transcription in Archaea

Jan-Peter Daniels[1], Steven Kelly[1,2,3], Bill Wickstead[1] and Keith Gull[1,*]

1) Sir William Dunn School of Pathology
University of Oxford
South Parks Road
OX1 3RE Oxford, UK

2) Centre for Mathematical Biology
University of Oxford
24-29 St Giles'
OX1 3LB Oxford, UK

3) Oxford Centre for Interactive Systems Biology
Department of Biochemistry
University of Oxford
South Parks Road
OX1 3QU Oxford, UK

*) corresponding author
email: keith.gull@path.ox.ac.uk
phone: 0044 (0) 1865 285455
fax: 0044 (0) 1865 275501

Suppl. Figure 1: Comparison of the Pfam profile-hidden Markov model (HMM) for Elf1 (PF05129.5; x-axis) and the Elf1 HMM used in this study (y-axis). Elf1 homologues identified in this study (red) and non-Elf1 sequences (black) are indicated. The histograms show the distribution of E-values of the sequences for both searches.

Suppl. Figure 2: Alignment of archaeal histones. The N- and C-termini of the alignment were trimmed. NCBI/Genbank accession numbers are displayed on the left. *Thermofilum pendens, Caldivirga maquilingensis* and Candidatus *Korarchaeum cryptofilum* sequences are marked with a yellow bar. Residues that are identical or similar to the consensus are shown with a blue or cyan background, respectively.

Suppl. Table 1: Sources of archaean predicted proteomes used in this study.

Suppl. Table 2: Sources of eukaryotic predicted proteomes used in this study.

Suppl. Table 3: Accession numbers of archaean Elf1 orthologues.

Suppl. Table 4: Accession numbers and predicted proteome IDs of eukaryotic Elf1 orthologues.

Suppl. Table 5: Sources of genomes (DNA sequence) used in this study.

Suppl. Figure 1

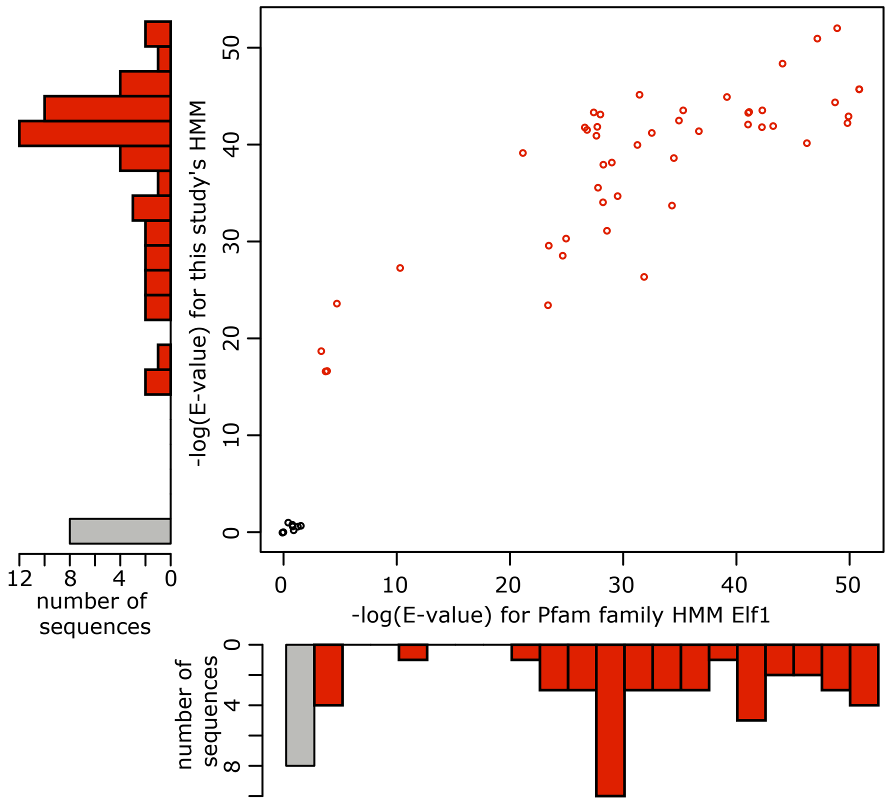

Suppl. Figure 2

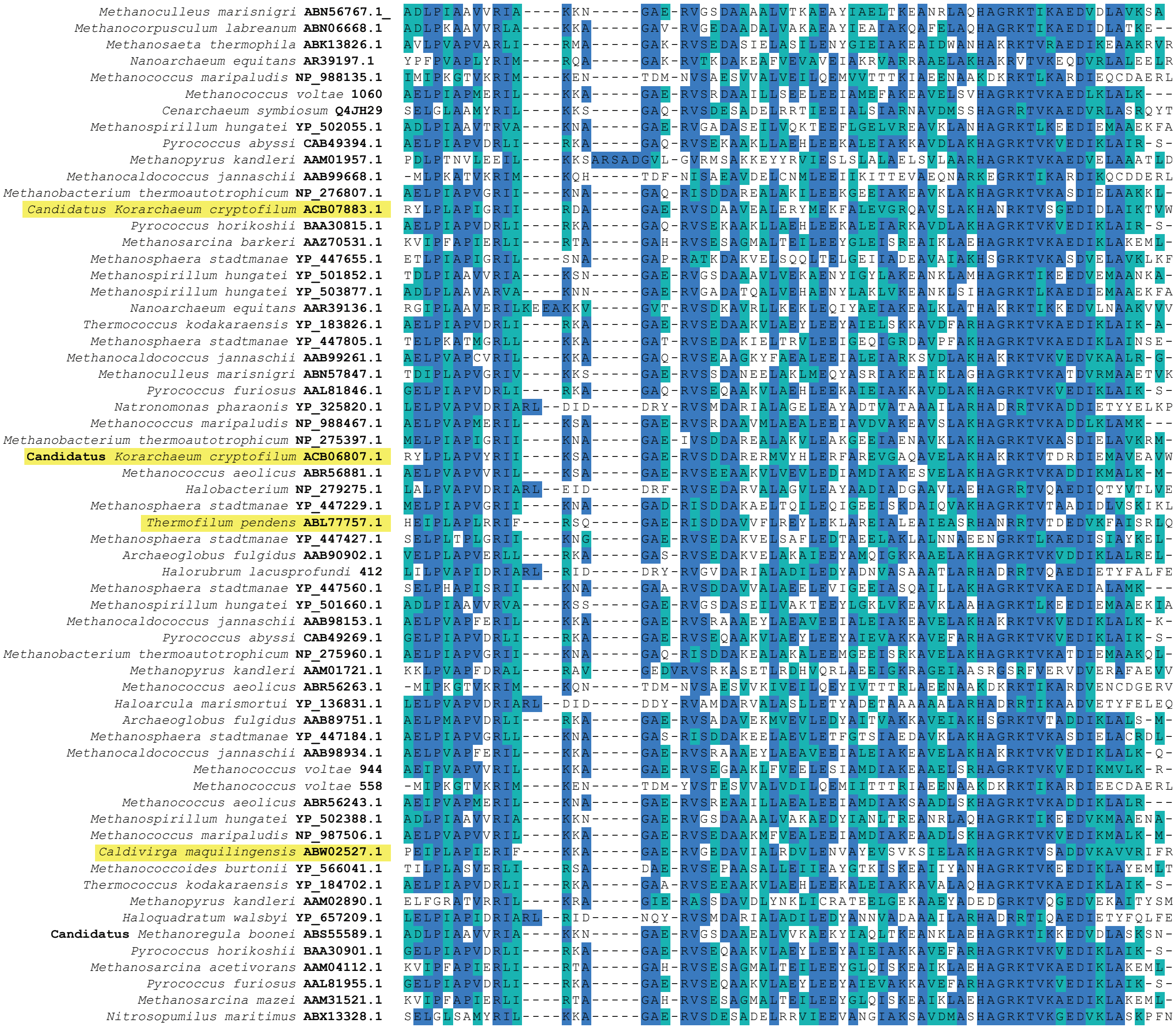

Suppl. Table 1: Sources of archaean predicted proteomes used in this study.

**Organism**	**Source**[1]	**Version**	**Web reference**
Aeropyrum pernix	Biotechnology Center	v1.0	http://www.tigr.org/
Archaeoglobus fulgidus	TIGR & UIUC	v1.0	http://www.tigr.org/
Caldivirga maquilingensis	US DOE JGI	v1.0	http://www.jgi.doe.gov/
Candidatus *Korarchaeum cryptofilum*	US DOE JGI	-	http://www.jgi.doe.gov/
Candidatus *Methanoregula boonei*	US DOE JGI	-	http://www.jgi.doe.gov/
Cenarchaeum symbiosum	MBARI	-	http://www.ebi.ac.uk/
Ferroplasma acidarmanus	US DOE JGI	-	http://www.jgi.doe.gov/
Haloarcula marismortui	UMBI/COMB	v16.0	http://www.tigr.org/
Halobacterium sp.	University of Massachusetts-Amherst, University of Washington and Institute for Systems Biology	v1.0	http://www.tigr.org/
Haloquadratum walsbyi	Max Planck Institute	v21.0	http://www.tigr.org/
Halorubrum lacusprofundi	US DOE JGI	-	http://www.jgi.doe.gov/
Ignicoccus hospitalis	US DOE JGI	-	http://www.jgi.doe.gov/
Metallosphaera sedula	US DOE JGI	-	http://www.jgi.doe.gov/
Methanothermobacter thermautotrophicus	Genome Therapeutics/Ohio State University	v1.0	http://www.tigr.org/
Methanococcoides burtonii	US DOE JGI	-	http://www.jgi.doe.gov/
Methanococcus aeolicus	US DOE JGI	-	http://www.jgi.doe.gov/
Methanocaldococcus jannaschii	TIGR & UIUC	v1.0	http://www.tigr.org/
Methanococcus maripaludis	US DOE JGI	-	http://www.jgi.doe.gov/
Methanococcus voltae	US DOE JGI	-	http://www.jgi.doe.gov/
Methanocorpusculum labreanum	US DOE JGI	-	http://www.jgi.doe.gov/
Methanoculleus marisnigri	US DOE JGI	-	http://www.jgi.doe.gov/
Methanopyrus kandleri	Fidelity Systems, Inc.	v8.0	http://www.tigr.org/
Methanosaeta thermophila	US DOE JGI	-	http://www.jgi.doe.gov/
Methanosarcina acetivorans	Broad institute	-	http://www.broad.mit.edu/
Methanosarcina barkeri	US DOE JGI	-	http://www.jgi.doe.gov/
Methanosarcina mazei	Goettingen Genomics Laboratory /Integrated Genomics Inc.	v8.0	http://www.tigr.org/
Methanosphaera stadtmanae	Goettingen Genomics Laboratory|Max-Planck-Institute, Marburg	v20.0	http://www.tigr.org/
Methanospirillum hungatei	US DOE JGI	-	http://www.jgi.doe.gov/
Nanoarchaeum equitans	Diversa	v15.0	http://www.tigr.org/
Natronomonas pharaonis	Max-Planck-Institute	v18.0	http://www.tigr.org/
Nitrosopumilus maritimus	US DOE JGI	-	http://www.jgi.doe.gov/
Picrophilus torridus	University of Goettingen/TU Hamburg-Harburg/Georg-August-Univ Goettingen	v16.0	http://www.tigr.org/
Pyrobaculum aerophilum	Caltech/UCLA	v7.0	http://www.tigr.org/
Pyrobaculum arsenaticum	US DOE JGI	-	http://www.jgi.doe.gov/
Pyrobaculum calidifontis	US DOE JGI	-	http://www.jgi.doe.gov/
Pyrobaculum islandicum	US DOE JGI	-	http://www.jgi.doe.gov/
Pyrococcus abyssi	Genoscope	v1.0	http://www.tigr.org/
Pyrococcus furiosus	Univ of Utah/Univ of Maryland	v7.0	http://www.tigr.org/
Pyrococcus horikoshii	MITI & Univ of Tokyo	v1.0	http://www.tigr.org/
Staphylothermus marinus	US DOE JGI	-	http://www.jgi.doe.gov/
Sulfolobus acidocaldarius	Epidauros Biotechnologie AG|Univ of Copenhagen	v20.0	http://www.tigr.org/
Sulfolobus solfataricus	European Union/Canadian Bioinformatics Resource	v3.0	http://www.tigr.org/
Sulfolobus tokodaii	Biotechnology Center	v7.0	http://www.tigr.org/
Thermococcus kodakaraensis	Kyoto University/Kwansei Gakuin University	v17.0	http://www.tigr.org/
Thermofilum pendens	US DOE JGI	-	http://www.jgi.doe.gov/
Thermoplasma acidophilum	Max Planck Institute for Biochemistry	v1.0	http://www.tigr.org/
Thermoplasma volcanium	AIST	v1.0	http://www.tigr.org/
Thermoproteus neutrophilus	US DOE JGI	-	http://www.jgi.doe.gov/

[1]) *Source of data does not necessarily correlate with sequencing centre(s). See individual projects for full acknowledgements of participants.*

Suppl. Table 2: Sources of eukaryotic predicted proteomes used in this study.

Organism	Source[1]	Version	Web reference
Arabidopsis thaliana	TAIR	TAIR7	www.arabidopsis.org/
Caenorhabditis elegans	WormBase	WS179	www.sanger.ac.uk/Projects/C_elegans/WORMBASE/
Chlamydomonas reinhardtii	US DOE JGI	v3.0	www.jgi.doe.gov/
Cryptosporidium parvum	CryptoDB	v3.4	www.cryptodb.org/
Cyanidioschyzon merolae	C.merolae genome project	-	merolae.biol.s.u-tokyo.ac.jp/
Dictyostelium discoideum	dictyBase	05-20-2007	dictybase.org/
Drosophila melanogaster	ENSEMBL	BDGP4.3, 46.43b	www.ensembl.org/info/data/
Entamoeba histolytica	geneDB	17102005	www.genedb.org/
Giardia lamblia	GiardiaDB	April 17, 2007	www.mbl.edu/Giardia
Homo sapiens	VEGA	Jun2006	vega.sanger.ac.uk/
Leishmania major	geneDB	v5.2	www.genedb.org/
Monosiga brevicollis	JGI	v1.0	www.jgi.doe.gov/
Naegleria gruberi	JGI	v1.0	www.jgi.doe.gov/
Paramecium tetraurelia	ParameciumDB	v1.11	paramecium.cgm.cnrs-gif.fr/
Phaeodactylum tricornutum	JGI	v2.0	www.jgi.doe.gov/
Physcomitrella patens	US DOE JGI	v1.1	www.jgi.doe.gov/
Phytophthora sojae	US DOE JGI	v1.1	www.jgi.doe.gov/
Phytophtora ramorum	US DOE JGI	v1.1	www.jgi.doe.gov/
Plasmodium falciparum	PlasmoDB	v5.3	www.plasmodb.org/
Saccharomyces cerevisiae	ENSEMBL	SGD1.01.46	www.ensembl.org/info/data/
Schizosaccharomyces pombe	Sanger	-	www.sanger.ac.uk/Projects/S_pombe
Takifugu rubripes	ENSEMBL	Assembly4, 46	www.ensembl.org/info/data/
Tetrahymena thermophila	TIGR	10/24/2006	www.tigr.org/tdb/e2k1/tta1/
Thalassiosira pseudonana	US DOE JGI	v3.0	www.jgi.doe.gov/
Theileria annulata	GeneDB	v4	www.genedb.org/
Toxoplasma gondii	ToxoDB	v4.1	www.toxodb.org/
Trichomonas vaginalis	TIGR	20050331	www.tigr.org/tdb/e2k1/tvg/
Trypanosoma brucei	geneDB	v4	www.genedb.org/

[1]) Source of data does not necessarily correlate with sequencing centre(s). See individual projects for full acknowledgements of participants.

Suppl. Table 3: Accession numbers of archaean Elf1 orthologues.

Organism	NCBI/Genbank reference
Aeropyrum pernix	BAA79593.2
Caldivirga maquilingensis	ABW01761.1
Candidatus *Korarchaeum cryptofilum*	ACB08272.1
Ignicoccus hospitalis	ABU81609.1
Metallosphaera sedula	ABP96427.1
Pyrobaculum aerophilum	AAL64635.1
Pyrobaculum arsenaticum	ABP51345.1
Pyrobaculum calidifontis	ABO08409.1
Pyrobaculum islandicum	ABL87622.1
Staphylothermus marinus	ABN70090.1
Sulfolobus acidocaldarius	YP_255398.1
Sulfolobus solfataricus	AAK40600.1
Sulfolobus tokodaii	BAB65278.1
Thermofilum pendens	NA
Thermoproteus neutrophilus	ACB40598.1

Suppl. Table 4: Accession numbers and predicted proteome IDs of eukaryotic Elf1 orthologues.

Organism	NCBI/Genbank reference
Arabidopsis thaliana	NP_568654.1
Caenorhabditis elegans	NP_496983.1
Chlamydomonas reinhardtii	XP_001691592.1
Cryptosporidium parvum	XP_626150.1
Cyanidioschyzon merolae	CMO247C
Dictyostelium discoideum	XP_637365.1
Drosophila melanogaster	NP_001104426.1; NP_996099.1
Giardia lamblia	XP_001709420.1
Homo sapiens	EAW84230.1; OTTHUMP00000077650
Leishmania major	XP_001682840.1; XP_001682841.1
Naegleria gruberi	5729
Paramecium tetraurelia	XP_001346933.1; XP_001458266.1; XP_001450964.1
Phaeodactylum tricornutum	XP_002183971.1
Physcomitrella patens	XP_001765330.1; XP_001763568.1
Phytophthora sojae	158411
Phytophtora ramorum	93621
Plasmodium falciparum	XP_001352018.1
Saccharomyces cerevisiae	NP_012762.1
Schizosaccharomyces pombe	NP_594786.1
Takifugu rubripes	SINFRUP00000161665
Tetrahymena thermophila	XP_001030656.1
Thalassiosira pseudonana	XP_002292763.1
Theileria annulata	XP_955243.1
Trichomonas vaginalis	XP_001305861.1
Trypanosoma brucei	XP_001218865.1; XP_001218870.1

Suppl. Table 5 (part 1 of 3): Sources of genomes (DNA sequence) used in this study.

**genome/plasmid**	**NCBI**	**Source**[1]	**Web reference**
Aeropyrum pernix	NC_000854.2	NITE	http://www.bio.nite.go.jp/dogan/MicroTop?GENOME_ID=ape_G1
Archaeoglobus fulgidus	NC_000917.1	TIGR	http://www.tigr.org/tigr-scripts/CMR2/GenomePage3.spl?database=gaf
Caldivirga maquilingensis	NC_009954.1	US DOE Joint Genome Institute	http://genome.jgi-psf.org/draft_microbes/calma/calma.home.html
Candidatus Korarchaeum cryptofilum	NC_010482.1	US DOE Joint Genome Institute	http://www.jgi.doe.gov/
Candidatus Methanoregula boonei	NC_009712.1	US DOE Joint Genome Institute	http://genome.jgi-psf.org/finished_microbes/metbo/metbo.info.html
Cenarchaeum symbiosum A	NS_000189.1	US DOE Joint Genome Institute	http://www.mbari.org/
Cenarchaeum symbiosum B	NS_000190.1	US DOE Joint Genome Institute	http://www.mbari.org/
Ferroplasma acidarmanus	JGI	US DOE Joint Genome Institute	http://genome.jgi-psf.org/ferac/ferac.info.html
Haloarcula marismortui chr I	NC_006396.1	Center of Marine Biotechnology	http://zdna2.umbi.umd.edu/
Haloarcula marismortui chr II	NC_006397.1	Center of Marine Biotechnology	http://zdna2.umbi.umd.edu/
Haloarcula marismortui pNG100	NC_006389.1	Center of Marine Biotechnology	http://zdna2.umbi.umd.edu/
Haloarcula marismortui pNG200	NC_006390.1	Center of Marine Biotechnology	http://zdna2.umbi.umd.edu/
Haloarcula marismortui pNG300	NC_006391.1	Center of Marine Biotechnology	http://zdna2.umbi.umd.edu/
Haloarcula marismortui pNG400	NC_006392.1	Center of Marine Biotechnology	http://zdna2.umbi.umd.edu/
Haloarcula marismortui pNG500	NC_006393.1	Center of Marine Biotechnology	http://zdna2.umbi.umd.edu/
Haloarcula marismortui pNG600	NC_006394.1	Center of Marine Biotechnology	http://zdna2.umbi.umd.edu/
Haloarcula marismortui pNG700	NC_006395.1	Center of Marine Biotechnology	http://zdna2.umbi.umd.edu/
Halobacterium sp.	NC_002607.1	University of Massachusetts-Amherst, University of Washington	http://zdna2.umbi.umd.edu/~haloweb/
Halobacterium sp. pNRC100	NC_001869.1	University of Massachusetts-Amherst, University of Washington	http://zdna2.umbi.umd.edu/~haloweb/
Halobacterium sp. pNRC200	NC_002608.1	University of Massachusetts-Amherst, University of Washington	http://zdna2.umbi.umd.edu/~haloweb/
Haloquadratum walsbyi	NC_008212.1	Max Planck Institute	
Haloquadratum walsbyi PL47	NC_008213.1	Max Planck Institute	
Halorubrum lacusprofundi chr 1	NC_012029.1	US DOE Joint Genome Institute	http://genome.jgi-psf.org/draft_microbes/halla/halla.info.html
Halorubrum lacusprofundi chr 2	NC_012028.1	US DOE Joint Genome Institute	http://genome.jgi-psf.org/draft_microbes/halla/halla.info.html
Halorubrum lacusprofundi pHLAC01	NC_012030.1	US DOE Joint Genome Institute	http://genome.jgi-psf.org/draft_microbes/halla/halla.info.html
Ignicoccus hospitalis	NC_009776.1	US DOE Joint Genome Institute	http://genome.jgi-psf.org/draft_microbes/ign_k/ign_k.home.html
Metallosphaera sedula	NC_009440.1	US DOE Joint Genome Institute	http://genome.jgi-psf.org/draft_microbes/metse/metse.info.html
Methanothermobacter thermautotrophicus	NC_000916.1	Genome Therapeutics Corporation	http://www.genomecorp.com/
Methanothermobacter thermautotrophicus pFV1	NC_001336.1	Agriculture Univ of Wageningen	

Suppl. Table 5 (part2 of 3): Sources of genomes (DNA sequence) used in this study.

genome/plasmid	NCBI	Source[1]	Web reference
Methanothermobacter thermautotrophicus pFZ1	NC_001337.1	Agriculture Univ of Wageningen	
Methanothermobacter thermautotrophicus pME2001	NC_002125.1	Molekulargenetik FB Biologie Philipps-Univesitaet, Marburg	
Methanothermobacter thermautotrophicus pME2200	NC_000905.1	Swiss Federal Institute of Technology, ETH, Institute of Microbiology	
Methanococcoides burtonii	NC_007955.1	US DOE Joint Genome Institute	http://genome.jgi-psf.org/draft_microbes/metbu/metbu.home.html
Methanococcus aeolicus	NC_009635.1	US DOE Joint Genome Institute	http://genome.jgi-psf.org/finished_microbes/metae/metae.info.html
Methanocaldococcus jannaschii	NC_000909.1	TIGR	http://www.tigr.org/tigr-scripts/CMR2/GenomePage3.spl?database=arg
Methanocaldococcus jannaschii extra-chr 1	NC_001732.1	TIGR	http://www.tigr.org/tigr-scripts/CMR2/GenomePage3.spl?database=arg
Methanocaldococcus jannaschii extra-chr 2	NC_001733.1	TIGR	http://www.tigr.org/tigr-scripts/CMR2/GenomePage3.spl?database=arg
Methanococcus maripaludis C5	NC_009135.1	US DOE Joint Genome Institute	http://genome.jgi-psf.org/draft_microbes/metmc/metmc.info.html
Methanococcus maripaludis C6	NC_009975.1	US DOE Joint Genome Institute	http://genome.jgi-psf.org/draft_microbes/metm6/metm6.home.html
Methanococcus maripaludis C7	NC_009637.1	US DOE Joint Genome Institute	http://genome.jgi-psf.org/draft_microbes/metm7/metm7.info.html
Methanococcus maripaludis pMMC501	NC_009136.1	US DOE Joint Genome Institute	http://genome.jgi-psf.org/draft_microbes/metmc/metmc.info.html
Methanococcus maripaludis pURB500	NC_001811.1	University of Georgia	http://www.uga.edu/
Methanococcus voltae	JGI	US DOE Joint Genome Institute	http://genome.jgi-psf.org/metvo/metvo.info.html
Methanocorpusculum labreanum	NC_008942.1	US DOE Joint Genome Institute	http://genome.jgi-psf.org/finished_microbes/metla/metla.home.html
Methanoculleus marisnigri	NC_009051.1	US DOE Joint Genome Institute	http://genome.jgi-psf.org/draft_microbes/metma/metma.info.html
Methanopyrus kandleri	NC_003551.1	Fidelity Systems	http://www.fidelitysystems.com/
Methanosaeta thermophila	NC_008553.1	US DOE Joint Genome Institute	http://genome.jgi-psf.org/finished_microbes/metth/metth.home.html
Methanosarcina acetivorans	NC_003552.1	Broad Institute	http://www.broad.mit.edu/annotation/microbes/methanosarcina/
Methanosarcina acetivorans pC2A	NC_002097.1	University of Illinois, Microbiology	
Methanosarcina barkeri chr 1	NC_007355.1	US DOE Joint Genome Institute	http://genome.jgi-psf.org/draft_microbes/metba/metba.home.html
Methanosarcina barkeri plasmid 1	NC_007349.1	US DOE Joint Genome Institute	http://genome.jgi-psf.org/draft_microbes/metba/metba.home.html
Methanosarcina mazei	NC_003901.1	Gottingen Genomics Laboratory	http://www.g2l.bio.uni-goettingen.de/projects/f_projects.html
Methanosphaera stadtmanae	NC_007681.1	University of Goettingen	http://www.g2l.bio.uni-goettingen.de/
Methanospirillum hungatei	NC_007796.1	US DOE Joint Genome Institute	http://genome.jgi-psf.org/finished_microbes/methu/methu.info.html
Nanoarchaeum equitans	NC_005213.1	DIVERSA	http://www.diversa.com/
Natronomonas pharaonis	NC_007426.1	Max Planck Institute	http://www.biochem.mpg.de/oesterhelt/genomics/intro_Napha.html

Suppl. Table 5 (part 3 of 3): Sources of genomes (DNA sequence) used in this study.

genome/plasmid	NCBI	Source[1]	Web reference
Natronomonas pharaonis PL23	NC_007428.1	Max Planck Institute	http://www.biochem.mpg.de/oesterhelt/genomics/intro_Napha.html
Natronomonas pharaonis PL131	NC_007427.1	Max Planck Institute	http://www.biochem.mpg.de/oesterhelt/genomics/intro_Napha.html
Nitrosopumilus maritimus	NC_010085.1	US DOE Joint Genome Institute	http://genome.jgi-psf.org/draft_microbes/nitma/nitma.info.html
Picrophilus torridus	NC_005877.1	University of Goettingen	http://www.g2l.bio.uni-goettingen.de/projects/c_proj_pt.html
Pyrobaculum aerophilum	NC_003364.1	UCLA/CalTech	http://informa.bio.caltech.edu/proj_summ_pyro.html
Pyrobaculum arsenaticum	NC_009376.1	US DOE Joint Genome Institute	http://genome.jgi-psf.org/finished_microbes/pyrar/pyrar.info.html
Pyrobaculum calidifontis	NC_009073.1	US DOE Joint Genome Institute	http://genome.jgi-psf.org/finished_microbes/pyrca/pyrca.home.html
Pyrobaculum islandicum	NC_008701.1	US DOE Joint Genome Institute	http://genome.jgi-psf.org/draft_microbes/pyris/pyris.info.html
Pyrococcus abyssi	NC_000868.1	Genoscope	http://www.genoscope.cns.fr/Pab/
Pyrococcus abyssi pGT5	NC_001773.1	Genoscope	http://www.genoscope.cns.fr/Pab/
Pyrococcus furiosus	NC_003413.1	Utah Genome Center	http://www.genome.utah.edu/
Pyrococcus horikoshii	NC_000961.1	NITE	http://www.bio.nite.go.jp/dogan/MicroTop?GENOME_ID=ot3
Staphylothermus marinus	NC_009033.1	US DOE Joint Genome Institute	http://genome.jgi-psf.org/finished_microbes/stama/stama.info.html
Sulfolobus acidocaldarius	NC_007181.1	Danish Archaea Centre	http://dac.molbio.ku.dk/
Sulfolobus solfataricus	NC_002754.1	European/Canadian Consortium	http://www-archbac.u-psud.fr/projects/sulfolobus/
Sulfolobus solfataricus pIT3	NC_005907.1	Univerity of Naples Federico II, Dipartimento di Chimica Biologica	
Sulfolobus tokodaii	NC_003106.2	NITE	http://www.bio.nite.go.jp/dogan/MicroTop?GENOME_ID=st__G1
Thermococcus kodakaraensis	NC_006624.1	Dragon Genomics	http://www.sbchem.kyoto-u.ac.jp/imanaka-lab/genome/index.html
Thermofilum pendens	NC_008698.1	US DOE Joint Genome Institute	http://genome.jgi-psf.org/draft_microbes/thepe/thepe.info.html
Thermofilum pendens pTPEN01	NC_008696.1	US DOE Joint Genome Institute	http://genome.jgi-psf.org/draft_microbes/thepe/thepe.info.html
Thermoplasma acidophilum	NC_002578.1	Max-Plank-Institute	http://www.biochem.mpg.de/baumeister/genome/home.html
Thermoplasma acidophilum pTA1	NC_008318.1	Tokyo University of Pharmacy and Life Science, Department of Molecular Biology	
Thermoplasma volcanium	NC_002689.2	National Institute of Advanced Industrial Science and Technology, Japan	http://www.aist.go.jp/RIODB/archaic/
Thermoproteus neutrophilus	NC_010525.1	US DOE Joint Genome Institute	http://genome.jgi-psf.org/draft_microbes/thene/thene.info.html

[1]) Source of data does not necessarily correlate with sequencing centre(s). See individual projects for full acknowledgements of participants.
